# Malignant DFFB isoform switching promotes leukemia survival in relapse pediatric T‐cell acute lymphoblastic leukemia

**DOI:** 10.1002/jha2.634

**Published:** 2022-12-30

**Authors:** Sabina Enlund, Indranil Sinha, Amanda Ramilo Amor, Shahrzad Shirazi Fard, Ekaterina Pokrovskaja Tamm, Qingfei Jiang, Vanessa Lundin, Anna Nilsson, Frida Holm

**Affiliations:** ^1^ Deparment of Women's and Children's Health Division of Pediatric Oncology and Surgery Karolinska Insitutet Stockholm Sweden; ^2^ Department of Oncology‐Pathology Karolinska Institutet Stockholm Sweden; ^3^ Division of Regenerative Medicine Department of Medicine Sanford Consortium for Regenerative Medicine University of California La Jolla California USA; ^4^ Moores Cancer Center La Jolla California USA; ^5^ Center for Hematology and Regenerative Medicine Department of Medicine Huddinge Karolinska Institutet Stockholm Sweden

**Keywords:** acute leukaemia, alternative mRNA splicing, apoptosis, CD34, childhood leukaemia, relapse

## Abstract

With modern treatment most children with acute lymphoblastic leukemia (ALL) survive without relapse. However, for children who relapse the prognosis is still poor, especially in children with T‐cell phenotype (T‐ALL) and remains the major cause of death. The exact mechanism of relapse is currently not known. While contribution of RNA processing alteration has been linked to other hematological malignancies, its contribution in pediatric T‐ALL may provide new insights. Almost all human genes express more than one alternative splice isoform. Thus, gene modulation producing a diverse repertoire of the transcriptome and proteome have become a significant molecular marker of cancer and a potential therapeutic vulnerability. To study this, we performed RNA‐sequencing analysis on patient‐derived samples followed by splice isoform‐specific PCR. We uncovered a distinct RNA splice isoform expression pattern characteristic for relapse samples compared to the leukemia samples from the time of diagnosis. We also identified deregulated splicing and apoptosis pathways specific for relapse T‐ALL. Moreover, patients with T‐ALL displayed pro‐survival splice isoform switching favoring pro‐survival isoforms compared to normal healthy stem cells. Cumulatively, pro‐survival isoform switching and DFFB isoform regulation of SOX2 and MYCN may play a role in T‐ALL proliferation and survival, thus serving as a potential therapeutic option.

## 1 INTRODUCTION

Acute lymphoblastic leukemia (ALL) is an aggressive hematological cancer in which proliferation of malignant lymphoid cells impair hematopoiesis and thus invade bone marrow, blood and extramedullary sites. In children, ALL remains the most common malignancy, where T‐cell ALL (T‐ALL) accounts for 10–15% of all cases. Although the overall survival probability is high, the cure‐rate remains low in the patients experiencing relapse. A critical factor complicating the prognosis and treatment is that T‐ALL exhibits the fewest genomic abnormalities among both hematological malignancies and solid tumors [1, 2], suggesting that additional molecular changes are required to hijack the normal developmental program.

Alternative processing of mRNA, a phenomenon mediated by the spliceosome complex, is essential for gene expression and regulation in higher organisms. Increased splicing complexity results in dramatically enlarged diversity in both the transcriptome and proteome. Several studies have shown that altered expression of such factors occurs in numerous different cancers and can be linked to malignant transformation [3, 4]. For example, CD44 have been shown to undergo extensive alternative mRNA splicing, resulting in several different isoform. In particularly CD44v3 has been implicated to play a role in the malignant progression from chronic phase to blast crisis in chronic myeloid leukemia [5]. Additionally, a recent study demonstrated a novel potential splicing‐targeted leukemia stem cell (LSC) eradication strategy for secondary acute myeloid leukemia which reduced the LSC burden and self‐renewal potential in serial transplantation assays. This landmark paper demonstrated a first step in preventing disease relapse and other malignancies typified by splicing regulation [6]. With the development of deep sequencing technology, several malignancies have been associated with an abnormal splicing signature. Thus, changes in isoform patterns may be considered as new biomarker strategies and potential therapeutic targets.

Specifically, since the genetics of T‐ALL is highly heterogenous between patients, we hypothesized that variations in splicing could result in a functional disruption of individual ALL‐associated genes rather than in other oncogenic pathways. To test the above hypothesize, we investigated the splicing variability in 48 patients diagnosed with T‐ALL at the time of diagnosis and relapse, by using publicly available sequencing data (pre‐generated by the TARGET initiative). We observed alternative splicing induced isoform switching in several genes that harbor change‐of‐function characteristics depending on which isoform is expressed. In addition, we found that alternative splicing of DNA Fragmentation Factor subunit Beta (DFFB), which has been implicated to play a role in other malignancies [7, 8], generated two different protein coding isoforms, DFFB‐201 and DFFB‐204, which to date, has never been studied. Thus, consideration of alternative splicing places DFFB, for the first time, among the set of genes commonly altered in T‐ALL. Moreover, overexpression of DFFB drives increased expression of known proliferation genes SOX2 and YAP1, suggesting a broad impact of this splicing event on the cellular transcriptome.

Together, our analysis demonstrates the contribution of alternative splicing to gene dysregulation in T‐ALL, highlighting the importance of quantitatively assessing this aspect of gene expression in determining the prevalence of gene disruption in disease. Moreover, a frequent deregulation of such a pro‐survival gene, as DFFB, in the relapsed T‐ALL may suggest it as a potential therapeutic target.

## 2 METHODS

### 2.1 RNA sequencing analysis

30 diagnosis T‐ALL and 18 Relapse T‐ALL samples were selected for this analysis (Supplementary Table S1), samples are not longitudinal. RNA sequencing data (.BAM) files were downloaded from GDC data portal (https://portal.gdc.cancer.gov/projects/TARGET‐ALL‐P2). SeqMonk Mapped Sequence Data Analyzer (version 1.47.1) was used to analyse the dataset. To generate DESeq2 normalized value for each isoform following criteria was used in SeqMonk; DESeq2 stats filter on probes in “All Probes” where Relapse vs None (diagnosis) had a significance below 0.05 after Benjamimi and Hochberg correction. Quantitation was RNA‐Seq pipeline quantitation counting reads over exons as raw counts, assuming an opposing strand specific library.

For Gene‐Set Enrichment Analysis (GSEA), we merge the isoforms for each gene into a single measure. We used RNA‐seq quantitation pipeline with all the above‐mentioned criteria except we set Merge transcript isoforms = yes. GSEA V4.2.3 (build 10) version and KEGG gene set database (c2.cp.kegg.v7.5.symbols.gmt) was used for this analysis.

To analyse gene isoforms, sample sequences were downloaded from GDC data portal. GenomicDataComons package and a R script was used (R version 4.0.3). SeqMonk was used to generate FPKM values.

### 2.2 T‐ALL cell lines

T‐ALL cell lines CCL, Jurkat, and SUP‐T1 from ATCC were cultured in accordance with the recommended protocol. MOLT16 and SUP‐T11 (Leibniz Institute DSMZ) were cultured in suspension using IMDM medium (Gibco) supplemented with 10% fetal bovine serum (Sigma‐Aldrich) and 1% penicillin‐streptomycin (Thermo Fischer Scientific). Media renewal and passaging was performed every 2–4 days and cells were incubated at 37°C with 5% CO_2_.

### 2.3 Primary patient sample collection and preparation

Mononuclear cells from primary T‐ALL samples were obtained from patients at Karolinska University Hospital Pediatric Oncology (**Ethical permission: dnr 2019‐00427; dnr 2021–02718**) after oral and written informed consent. CD34+ cord blood samples were purchased from BioNordika, Stockholm, Sweden. Samples underwent positive CD34 selection using EasySep Human CD34 positive Selection kit II (Stem Cell Technologies).

### 2.4 Splice isoform and qRT‐PCR analysis

Total RNA from CD34+ cells were extracted with RNeasy Mini Kit (Qiagen). We used 500 ng of template RNA for reverse transcription to cDNA with SuperScript III First Strand Synthesis SuperMix for qRT‐PCR (Invitrogen). qRT‐PCR was performed using SYBR GreenER qPCR SuperMix for iCycler (Invitrogen). Target mRNA was normalized to hypoxanthine phosphoribosyltranferase (HPRT) and relative fold difference in expression was calculated via the delta‐delta CT method. Primer sequences can be found in Supplementary Table S2.

### 2.5 Immunoblot

Sample preparation, SDS‐PAGE and Western blot analysis were performed as described previously [9, 10]. The following antibodies were used in this study: DFFB Mouse anti‐Human monoclonal antibody (Invitrogen, MA517214, 1:2000), Recombinant Anti‐beta Tubulin rabbit monoclonal antibody (Abcam, ab108342, 1:2000), IRDye® 800CW Donkey anti‐Rabbit IgG Secondary Antibody (LI‐COR Biosciences, 926–32212, 1:20000) and IRDye® 680RD Donkey anti‐Mouse IgG Secondary Antibody (LI‐COR Biosciences, 926–68072, 1:20000).

### 2.6 DFFB overexpression

**Endotoxin‐free plasmid DNA purification**. DH5α competent cells (Invitrogen) were transformed by heat‐shock as previously described [9, 10] using DFFB Human Tagged ORF Clone (Origene, RG237731) or pCMV6‐AC‐GFP Mammalian Expression Vector (Origene, PS100010).

Plasmid DNA purification was performed using Nucleobond^®^ Xtra Maxi EF kit (Macherey‐Nagel) and plasmid concentration using Nucleobond^®^ Finalizer (Macherey‐Nagel).

**Transfections**. Cell‐line‐specific Nucleofector Kits (Lonza) used were and transfections were performed in accordance to manufacturers protocols. Nucleofection efficiency was determined by RT‐qPCR 24 hours post transfection.

### 2.7 Qiagen array

RNA extracted 24 h post transfection was used to perform RT^2^ Profiler PCR Arrays (Qiagen) pre‐coated with primers for genes associated with apoptosis or cancer stem cells. Master mix containing 500 ng cDNA was used per array. Plates were analyzed using the Bio‐Rad CFX Connect Real‐Time PCR Detection System (Bio‐Rad).

### 2.8 Confluency assay

80 × 10^3^ MOLT16 cells were seeded in 200 μl medium/well in a 96 well plate, and the edges were filled with medium to avoid edge effects. Plates were scanned by an IncuCyte S3 Live® Cell Analysis System (Essen Bioscience) every 3 hours for 72 hours. The proliferation was determined by measuring the cell confluency. Each treatment was done in 2–3 replicates.

### 2.9 Statistical analysis

For RNA‐sequencing Qlucore Omics Explorer (version:3.8) was used to calculate p‐values, FDRs and fold change values. Hierarchical clustering method in Qlucore was used to generate different heatmaps. All other data were compared using either unpaired t‐test with Welch's correlation or Pearson correlation coefficients, two‐tailed with 95% confidence interval.

## 3 RESULTS

### 3.1 Splicing deregulation distinguishes T‐ALL at relapse compared to time of diagnosis

**FIGURE 1:**
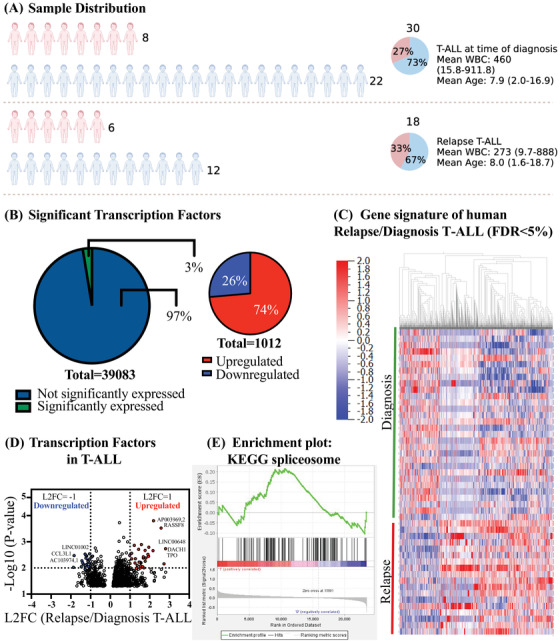
Gene signature of relapse T‐ALL. (A) Schematic overview of included patients. Samples were distributed among time of diagnosis (*n* = 30) and relapse (*n* = 18). Only bone marrow samples were included in this co‐hort. Distribution male:female is 22:8 for time of diagnosis, with a mean WBC of 460 and mean age of 7.9. For relapse, the male:female distribution is 12:6, mean WBC of 273 and mean age of 8. (B) Distribution of significant transcription factors in patients with T‐ALL, at the time of diagnosis and relapse (left pie‐chart), and the distribution between significant up‐ and downregulated transcription factors (right pie‐chart). (C) Log 2 fold change (L2FC) and *p* values were computed from gene expression data (FPKM, relapse/diagnosis). Profiles of all differentially expressed genes (*p* < 0.05) are shown. (D) Volcano plot analysis of all 1012 significantly expressed transcription factors in relapse versus diagnosis T‐ALL. L2FC was calculated for each transcription factor using normalized FPKM values. (E) GSEA spliceosome enrichment plot for relapse versus time of diagnosis in patients diagnosed with T‐ALL showing the profile running ES score and positions of gene set members on the ranked‐ordered list

To generate a comprehensive transcriptome expression map of human pediatric T‐ALL we included a total of 48 patients, consisting of bone‐marrow samples from 18 patients with relapsed T‐ALL and 30 samples from patients at time of diagnosis (Figure 1A). Sample inclusion criteria were based on their MLL‐status (negative), white blood count (WBC), age and tissue (Table 1). We performed whole gene, splice isoform, transcription factor and lncRNA analysis to generate a comprehensive map of distinct transcriptomic differences between these two patient groups. Notably, in pediatric T‐ALL samples from time of diagnosis versus relapse we identified only 3% significantly differentiated expressed genes, with most of the genes, 74%, being upregulated at relapse compared to at the time of diagnosis (Figure 1B). The gene signature is also distinctly different between the groups (Figure 1C). Differential gene expression analysis identified that inflammation‐responsive transcription factors, such as CCL3L1, characterized patients with relapse T‐ALL, along with deregulation of several key cancer regulators such as FAT3 [11], CFTR [12] and GLI3 [13] (Figure 1D, Figure SF1A). Notably, we identified increased expression of the tumor suppressor gene RASSF8 in patients at time of relapse (Figure 1D, Figure S1A,B, Supplementary Table S3). GSEA reveled disruption of vital stem cell regulatory pathways such as DNA replication in patients with relapse T‐ALL (Figure S1C). At the time of diagnosis inflammation‐associated pathways such as Graft versus Host Disease, Allograft Rejection and Viral Myocarditis was observed (Figure S1D). Notably, KEGG spliceosome pathway revealed distinct expression changes between time of diagnosis and relapse indicating a differential splicing regulation during relapse (Figure 1E).

### 3.2 Splice isoform signature of human pediatric T‐ALL

**FIGURE 2:**
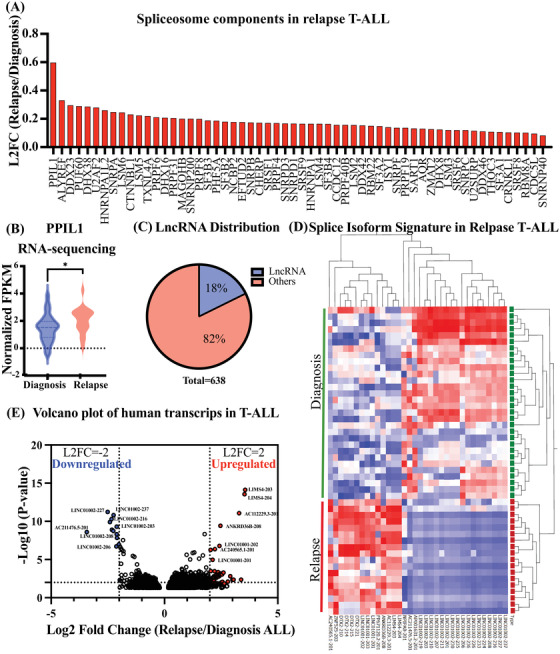
Splice isoform signature of relapse T‐ALL. Whole transcriptome sequencing results from 30 patients with T‐ALL at time of diagnosis and 18 patients with relapse T‐ALL. Only samples from bone marrow were analyzed. Gene and isoform expression data in FPKM were used to calculated average log 2 fold change (L2FC) and *p* values and FDR corrections. (A) Waterfall plot showing average L2FC of KEGG spliceosome components comparing RNA‐Seq data from T‐ALL relapse versus diagnosis samples. (B) RNA‐Seq‐based analysis (normalized FPKM) showing significantly increased expression of spliceosome component PPIL1 in patients with relapsed T‐ALL (*p* < 0.05). (C) LncRNA distribution of significantly differentially expressed genes (*n* = 638) in patients with relapse T‐ALL compared to time of diagnosis (data excluding novel transcripts). (D) Splice isoform heat map of top 75 differentially expressed isoforms between relapse T‐ALL and time of diagnosis are shown. (E) Volcano plot analysis of all significantly expressed transcripts (*n* = 6041) in relapse versus time of diagnosis are shown. L2FC was calculated for each transcript using FPKM values.

To determine if T‐ALL relapse evolves due to splicing deregulation, we performed pathway specific analysis of RNA‐seq data that reveled significant alterations in gene expression of many splicing factors in patients with relapse T‐ALL, including upregulation of Peptidyl‐Prolyl Isomerase Like‐1 (PPIL1), ALYREF and members of the SF3 family such as SF3B3, SF3B2, SF3B4, and SF3A1 (Figure 2A, Supplementary Figure S2A). Among the upregulated transcripts SF3B2, PUF60, and PHF5A are components of the U2 complex that promotes splicing (Figure 2A). ALYREF, ISY1, and RBM8A participates in the exon junction complex (Figure 2A). The top upregulated gene in the spliceosome complex, PPIL1, (Figure 2A‐B) joins the major spliceosome complex formation and remains there until splicing is complete [14].

Together, these data suggest that spliceosome disruption is prevalent in T‐ALL and may drive splicing alterations of stem cell regulatory genes contributing to LSC generation, thus leading to relapse. Moreover, long non‐coding RNAs (lncRNAs) have emerged as key determinants of both mouse [15] and human [6] hematopoietic stem cell fate commitment and alternative splicing. LncRNA profiling revealed that 18% of all differentially expressed genes are LncRNAs. In particular, we identified a relapse T‐ALL specific lncRNA, SENCR (Figure S2B), known to interact with TAL1, a common master transcription factor in T‐ALL, through FLI1 [16].

Next, we evaluated splice isoform profiles in T‐ALL at time of diagnosis versus at relapse. Utilizing an isoform‐specific alignment algorithm, we identified a splice isoform signature of relapse T‐ALL that were distinct from samples from the diagnosis time point (Supplementary Table S4, Figure 2D). 3% of all transcripts were significantly differentially expressed between patients at time of diagnosis and patients at relapse, where 81% were significantly upregulated (Figure S2C). Upregulated isoforms during relapse included isoforms of transcription factors, LncRNA and zinc finger proteins (Figure 2E), indicative of a prominent spliceosomal contribution to developing relapse. These whole gene and splice isoform expression signatures of human pediatric T‐ALL identify stem cell and splicing pathways that are deregulated in T‐ALL relapse patients.

### 3.3 Relapse T‐ALL is typified by pro‐survival splice isoform switching

**FIGURE 3:**
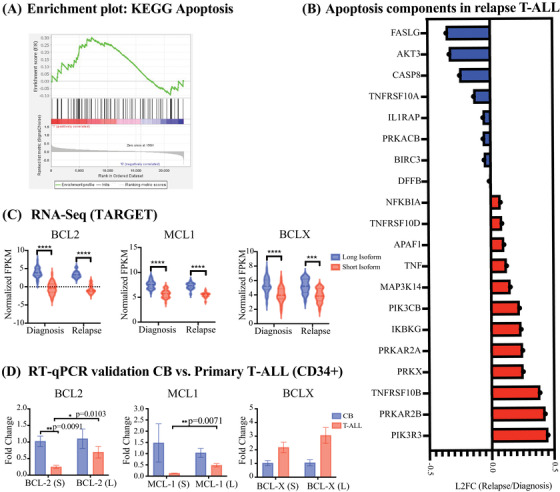
Splice switching distinguishes high risk T‐ALL. Gene and isoform expression data in FPKM were obtained from TARGET data‐set and obtained from patients at time of diagnosis with T‐ALL and relapse T‐ALL. GSEA was performed using all KEGG pathways. (A) GSEA Apoptosis enrichment plot for relapse versus primary patients diagnosed with T‐ALL showing the profile running ES score and positions of gene set members on the ranked‐ordered list. (B) Waterfall plot showing average L2FC of KEGG apoptosis components comparing RNA‐Seq data from patients at diagnosis and relapsed T‐ALL. (C) RNA‐Seq‐based analysis (normalized FPKM) showing increased expression of pro‐survival isoforms (long isoform) of BCL‐2, MCL1, and BCL‐X in (***p < 0.001, ****p < 0.0001). D) Splice isoform‐specific qRT‐PCR quantification showing upregulation of long pro‐survival isoform of BCL2 and MCL1, but not BCLX in CD34+ LSC compared to CB (*p < 0.05, **p < 0.01)

GSEA of samples from patients with relapse T‐ALL revealed that apoptosis was a disrupted KEGG gene set compared to the time of diagnosis (Figure 3A). Among the dysregulated genes, AKT3 and two members of the Phosphatidylinositol‐3‐kinase family PIK3R3 and PIK3CB were identified (Figure 3B). Long isoforms of the Bcl2 family of apoptosis regulatory genes, including BCL2, BCL2L1 (BCLX), BCL2A1 (BFL1), and MCL1, promote cell survival, while short isoforms are pro‐apoptotic [17] (Figure S3A). GSEA revealed that apoptosis regulators were among the enriched gene sets in samples from patients with relapse T‐ALL compared to samples from patients at T‐ALL diagnosis (Figure 3A). Expression of the long pro‐survival isoform, BCL2‐L, MCL1‐L and BCL2L1 (BCLX‐L), was upregulated in samples both from patient at diagnosis and relapse, while only BCL2 (whole gene) were significantly differentially expressed (Figure 3C and Figure S3B). Hence, pro‐survival splice isoform switching may have clinical utility in predicting malignant transformation. To validate these results, we performed isoform‐RT‐qPCR in T‐ALL cell lines (Figure S3C), revealing an upregulation of the long isoform of MCL1 and BCLX. Interestingly, in CD34+ LSC‐enriched fractions from three T‐ALL patient samples and three normal CD34+ cord blood samples validated a significant upregulation of the long pro‐survival isoforms BCL2 and MCL1, but no significant difference in the isoform expression of BCLX (Figure 3D). Hence, pro‐survival isoform switching may have clinical utility in early detection of relapse.

### 3.4 DFFB isoform switching in high‐risk T‐ALL and its role in promoting leukemia proliferation and survival

**FIGURE 4:**
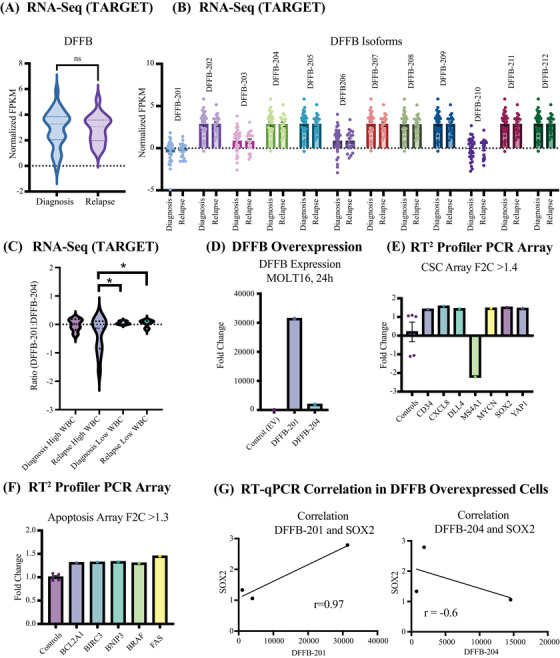
DFFB isoform switching promotes leukemia proliferation and survival. (A) RNA‐Seq‐based analysis (normalized FPKM) show no differential expression of DFFB in patients at the time of diagnosis compared to patients with relapse T‐ALL. (ns = not significant by two‐tailed, unpaired Student's t‐test). (B) RNA‐Seq‐based isoform analysis (normalized FPKM) of the 12 isoforms of DFFB in patients at diagnosis compared to patients with relapse T‐ALL. (C) Quantification of DFFB‐201/204 expression ratios (*p* < 0.05). Outlier excluded statistically in ratio analysis. (D) mRNA expression levels of DFFB‐201 and DFFB‐204 in T‐ALL cell line SUP‐T1 24 h post transfection. EV = Empty vector (Control). (E) Gene expression (fold change > 1.4) from QIAGEN RT^2^ profiler PCR cancer stem cell array. (F) Gene expression (fold change > 1.3) from QIAGEN RT^2^ profiler PCR apoptosis array. (G) Left panel: mRNA expression levels of DFFB‐201 positively correlates with SOX2 (*r* = 0.97) in SUP‐T1, Jurkat and MOLT16 T‐ALL cell line (Left panel) after DFFB overexpression. Right panel: DFFB‐204 negatively correlates with SOX2 (*r* = ‐0.6) in Jurkat, MOLT16 and SUP‐T1 T‐ALL cell line after DFFB overexpression. Correlation was calculated using Pearson correlation coefficients, two‐tailed with 95% confidence interval.

Furthermore, we identified DNA fragmentation factor subunit beta (DFFB) to be highly expressed in T‐ALL cell lines (Figure S4A). Although the full‐length gene of DFFB did not display a significantly differential expression at the time of diagnosis compared to relapse T‐ALL (Figure 4A), it was recognized to undergo extensive alternative mRNA splicing. Thus, resulting in 12 different isoforms (Figure 4B), two of which are protein coding isoforms, one long, DFFB‐201, and one short, DFFB‐204. Interestingly, a ratio calculation of the long and short DFFB isoform (201/204) shows a larger span in isoform switching in high‐risk patients (> 100 WBC at diagnosis) compared to patient with low WBC (< 100) highlighting a dysregulated splicing pattern in high‐risk patients (Figure 4C). However, our isoform RT‐qPCR validation experiment show significant upregulation of the short isoform in CD34+ T‐ALL LSCs (Figure S2B). Taken together, this is indicative that DFFB isoforms may have clinical importance in patients with very high WBC at time of diagnosis, thus switching between the long and short isoform expression. To further investigate the role of DFFB, we transfected T‐ALL cell lines with a DFFB overexpression plasmid (Figure 4D, Figure S4C–E). The DFFB overexpressed cells where subjected to Qiagen RT^2^ Profiler PCR arrays for evaluation of stem cell specific marker expression (Figure 4E) and apoptosis specific marker expression (Figure 4F). Through the array, we could identify that an upregulation of DFFB results in an upregulation of LSC marker CD34, as well as genes involved in inflammatory response, including CXCL8 (also known as IL‐8). Interestingly, an upregulation of DLL4 was found. DLL4 is one of the most important regulators of NOTCH1, which is a driving oncogene in T‐ALL [18, 19]. Additionally, the proto‐oncogene MYCN was upregulated together with SOX2 and YAP1, both markers for stem cell self‐renewal, reprogramming and homeostasis, as well as mechanistically promoting proliferation, survival, invasion/metastasis, cancer stemness and drug resistance [20, 21]. In the apoptosis array (Figure 4F), we further identified a regulator of YAP1, BIRC3 [22]. Together with BCL2A1, BIRC3 also serves as an antiapoptotic gene. In addition to being important in apoptosis, the upregulated genes are also directly involved in the BCL2 family (BNIP3), cell growth (BRAF) and proliferation (FAS). Finally, the DFFB‐201 expression correlates with the long isoforms of MCL1 and BCLX, but not with BCL2 (Figure S4E). However, a positive correlation between DFFB (full length gene) and MYCN is seen in the TARGET dataset across all samples (Figure S4E) and affect proliferation rate (Figure S4F). Upon DFFB overexpression the long isoform of DFFB (DFFB‐201) positively correlates with SOX2, while a negative correlation of the short isoform of DFFB (DFFB‐204) is shown (Figure 4G).

Cumulatively, this data suggests that pro‐survival isoform switching of BCL2 family members together with DFFB isoform regulation may play a role in leukemia proliferation and survival and thus a potential therapeutic option with a pharmacological splicing modulatory compound.

## 4 DISCUSSION

Alternative mRNA splicing is a fundamental step in gene expression by producing transcriptome diversity in eukaryotic cells. Since alternative mRNA splicing is the main driver of biological diversity it is not surprising that it also plays a vital role in the characteristics of many cancers. Lately, studies have begun to address the role of alternative mRNA splicing and the unique RNA transcripts derived from individual gene loci on a larger scale. It has been demonstrated that different protein isoforms of single genes often behave more like distinct proteins rather than variants of the same gene [23]. Thus, it is essential to re‐frame our understanding of mRNA and although its importance has been proven in several studies, it has been largely neglected as a source of novel biomarkers and therapeutic targets for drug development. Here, we demonstrate that relapse T‐ALL have a distinct splicing pattern, typified by deregulation of spliceosomal components and regulators. A recent transcriptomic analysis of chronic lymphocytic leukemia cells reveled that mutations in the small ribonuclear protein U2 component SF3B, which is involved in 3´splice‐site recognition, resulted in increased levels of alternative 3′ splice‐site events [24]. Additionally, a recent re‐analysis of data from The Cancer Genome Atlas (TCGA) identified 119 genes that encode core spliceosome and splicing factors carry putative driver mutations across 33 different tumor types [25]. Together this highlights the extent to which such mutations affect cancer development.

Our comparative RNA‐Seq analyses demonstrate that T‐ALL patients display pro‐apoptotic splice isoform switching of BCL2 family members following the transition to relapse. Apoptosis is a well‐known process for growth and development of organisms and the ability to obtain immortality by escaping programed cell death is a lifeline for a cancer cell. There is a large body of work implicating the change‐of‐function of BCL‐family members based on the altered expression of the isoforms [6, 17], thus it is not surprising that T‐ALL follow a similar pattern. Additionally, we found a similar change‐of‐function role of another apoptosis gene, DFFB and its long and short isoforms. DFFB is a major intracellular nuclease involved in DNA fragmentation during apoptosis [26, 27]. DFFB have previously been described to play a role in persister cell mutagenesis and tumor relapse [28] and its under‐expression have been reported in numerous malignancies such as endometrial malignancies and glioblastoma [29, 30]. Additionally, it has been reported to interact with BCL2, which is a well‐known inhibitor of apoptosis, and have been linked to more aggressive and high‐grade cancers [31]. Importantly, DFFB interplay with SOX2 [20], YAP1 [22] and MYCN [32] provide a theoretical foundation of its role in survival and proliferation. Thus, providing important insights into aberrant RNA splicing that can lead to novel clinical strategies for treating pediatric T‐ALL.

## AUTHOR CONTRIBUTIONS

Sabina Enlund, Amanda Ramilo Amor, Frida Holm and Indranil Sinha collected and analysed the data. Frida Holm conceptualized the project, analyzed data and wrote the paper. Anna Nilsson and Ekaterina Pokrovskaja Tamm provided primary patient samples. Qingfei Jiang, Shahrzad Shirazi Fard and Vanessa Lundin contributed with scientific expertise and were involved in writing and reviewing the paper.

## CONFLICT OF INTEREST

The authors declare no conflict of interest.

## ETHICS STATEMENT

Mononuclear cells from primary T‐ALL samples were obtained from patients at Karolinska University Hospital Pediatric Oncology (Ethical permission: dnr 2019‐00427; dnr 2021‐02718) after oral and written informed consent. CD34+ cord blood samples were purchased from BioNordika, Stockholm, Sweden. Both oral and written informed consent was obtained.

## Supporting information

Supporting Information

Supporting Information

Supporting Information

Supporting Information

Supporting Information

Supporting Information

Supporting Information

Supporting Information

## Data Availability

The data pertinent to the findings are part based upon data generated by the Therapeutically Applicable Research to Generate Effective Treatments (TARGET) initiative, phs000218, managed by the NCI. The data used for this analysis are available at https://portal.gdc.cancer.gov/projects/TARGET‐ALL‐P2. Additional analysis of this report is available from corresponding author upon reasonable request.
